# Isolated lichen planus of lip: Diagnosis and treatment monitoring using dermoscopy

**DOI:** 10.1002/ccr3.1933

**Published:** 2018-11-22

**Authors:** Mahesh Mathur, Prakash Acharya, Alina Karki, Nisha KC, Jyoti Shah, Ayush Jha

**Affiliations:** ^1^ Department of Dermatology College of Medical Sciences Bharatpur Nepal

**Keywords:** dermoscopy, lichen planus, mucoscopy, oral lichen planus, wickham striae

## Abstract

Lichen planus (LP) of lip has chances of malignant transformation as it may be exposed to external trauma, smoking, and ultraviolet light. This case highlights the use of dermoscope as a quick noninvasive tool for the diagnosis of LP of lip and monitoring the response to treatment.

## 1 INTRODUCTION

Although studies have described the dermoscopic patterns of cutaneous lichen planus, reports regarding dermoscopic features of lichen planus of lip are lacking. We aim to highlight the use of dermoscope during both the diagnosis and treatment of the lichen planus of lip.

Lichen planus (LP) reveals specific dermoscopic patterns that may help in clinical diagnosis. These patterns include Wickham striae (WS) with peripheral dotted and linear vessels.^1^, ^2^ Although multiple studies have described the dermoscopic features of cutaneous LP, very few studies exist for dermoscopy of oral LP. To our knowledge, there is a single case report of dermoscopy of LP of the lower lip in the Korean language.^3^ In view of the risk of malignant transformation, early diagnosis and active early treatment are necessary.^4^ Only, a few studies have described isolated LP of the lips until now.^4^ We report this case describing dermoscopy of oral LP localized to the lower lip because this method may allow early diagnosis, monitoring of treatment, and may also result in increased patient compliance.

## 2 REPORT OF A CASE

**Figure 1:**
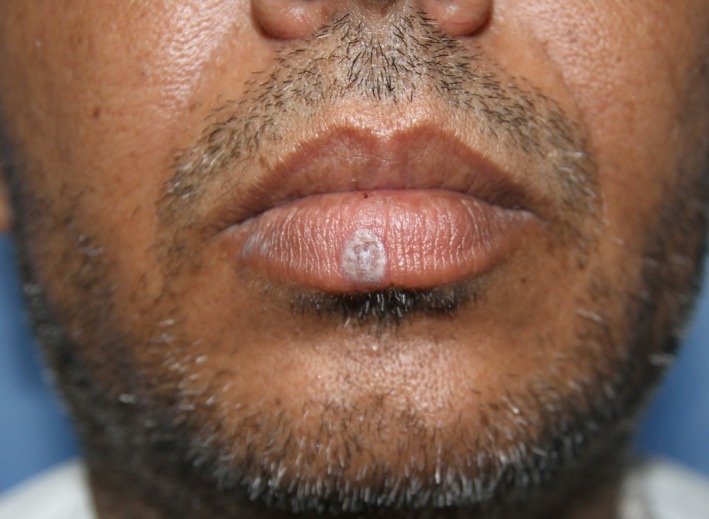
Clinically, whitish scaly plaques are seen over the medial and right lateral aspect of lower lip

**Figure 2:**
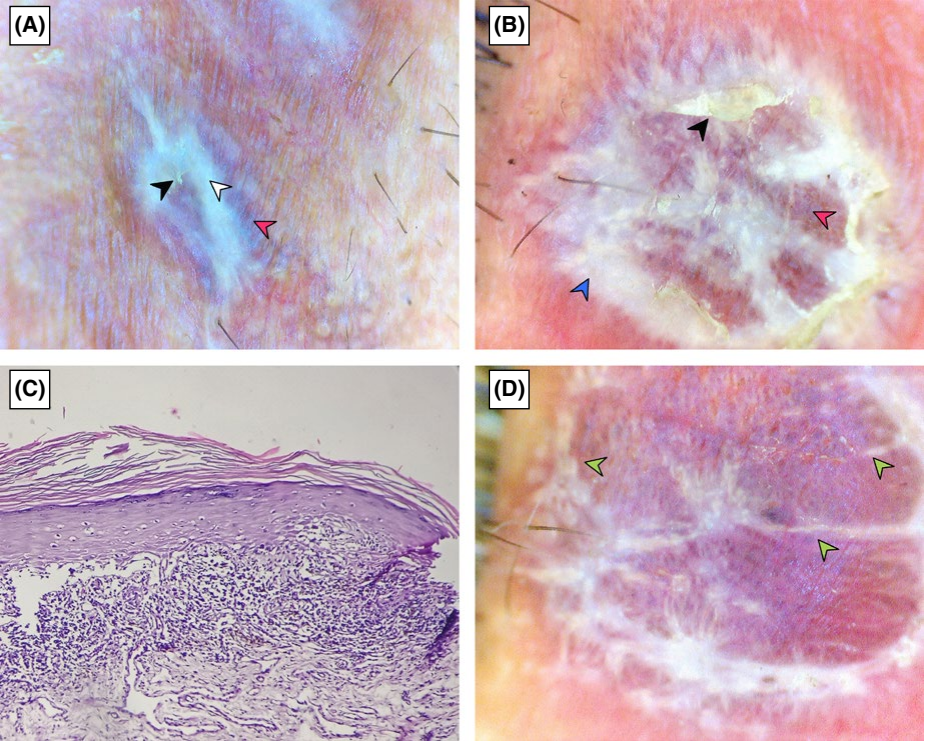
A and B, Dermoscopy (polarized dermoscopy, original magnification ×20) of lateral and medial lesions showing linear Wickham striae (white arrowhead) and circular Wickham striae (blue arrowhead), scaling (black arrowhead), and violaceous background (red arrowhead). C, Histopathology (hematoxylin‐eosin, original magnification ×40) showing hyperkeratosis, basal vacuolar degeneration, and prominent band‐like lymphocytic infiltrate. D, Dermoscopy of medial lesion after 14 days of treatment showing significantly reduced amount and thickness of Wickham striae (green arrowhead)

A 44‐year‐old male presented with a four‐month history of a raised and scaly plaque over the medial aspect of lower lip followed by the appearance of two similar lesions over the right lateral aspect of the same lip (Figure 1). Examination of skin, nails, and buccal mucosa revealed no abnormalities. He denied any previous treatments. Polarized light dermoscopy (Firefly Pro, MA, USA) over the lateral lesion showed WS in a linear pattern and medial lesion showed prominent WS in a circular pattern. Diffuse scaling and violaceous background were seen in dermoscopy of both lesions (Figures 2A,B).

A punch biopsy was performed from the lateral lesion and the histology showed hyperkeratosis, basal vacuolar degeneration, and prominent band‐like lymphocytic infiltrate (Figure 2C). Direct immunofluorescence test was negative for IgG, IgM, IgA, or C3 at the basement membrane zone. These features favored the diagnosis of LP, and the patient was prescribed topical betamethasone dipropionate 0.5 percent ointment. The medial lesion was re‐evaluated by dermoscopy on the 14th day which revealed a significant reduction in the amount and thickness of the WS and scaling was almost absent (Figure 2D). The same treatment was continued because a good response to treatment was noted.

## 3 DISCUSSION

Oral lichen planus (LP) most commonly affects the buccal mucosa, but the tongue, gingivae, floor of the mouth, and lips may also be affected.^5^ LP of lip should be differentiated from other forms of cheilitis like exfoliative cheilitis, lichen simplex chronicus, actinic cheilitis, discoid lupus erythematosus, pemphigus vulgaris, and herpes simplex.^6^ Since malignant transformation can occur, prompt treatment is necessary.^4^

Wickham striae are considered hallmark sign of LP which may appear as pearly whitish structures.^7^, ^8^ WS have not been described in any other conditions, and they are reported as constant dermoscopic finding of LP making them specific for LP.^8^ They may display several morphological patterns among which the reticular pattern is the most common.^7^ Other patterns that have been described include circular, linear, radial streaming, annular, round, leaf venation like, and starry sky patterns.^1^, ^2^, ^7^, ^8^ The appearance of WS has been attributed to the increase in granular cell layer in the epidermis by some authors and to the focal increase in the epidermal activity by others.^9^ WS are seen during active stages and disappear after treatment which could establish them as an activation marker for LP.^2^ In our patient, the dermoscopic finding of WS during diagnosis and their regression during treatment are consistent with the findings of earlier studies.

Multiple treatment modalities for the treatment of oral LP have been reported, including topical and oral corticosteroids, retinoids, azathioprine, griseofulvin, cyclosporin, imiquimod, mycophenolate, dapsone, tacrolimus, and chloroquine.^5^ Our patient showed a good response to topical betamethasone as demonstrated by the dermoscopic changes.

## 4 CONCLUSION

We conclude by saying that dermoscopy may be a useful noninvasive tool during diagnosis of LP of lip and monitoring of treatment as it allows closer visualization of lesions. The dermoscopic changes could be photographed using a computer and instantly shown to the patient. This could result in increased patient compliance and ultimately lead to successful treatment.

## CONFLICT OF INTEREST

None declared.

## AUTHOR CONTRIBUTION

MM and PA: collected clinical data and wrote the manuscript. AK and NK: contributed to patient evaluation, diagnosis, treatment, and follow‐up. JS and AJ: supervised and reviewed the manuscript. All authors read and approved the final version of the manuscript.
